# Implementing eHealth Technology to Address Gaps in Early Infant Diagnosis Services: Qualitative Assessment of Kenyan Provider Experiences

**DOI:** 10.2196/mhealth.9725

**Published:** 2018-08-22

**Authors:** Catherine Wexler, Melinda Brown, Emily A Hurley, Martin Ochieng, Kathy Goggin, Brad Gautney, May Maloba, Raphael Lwembe, Samoel Khamadi, Sarah Finocchario-Kessler

**Affiliations:** ^1^ Department of Family Medicine University of Kansas Medical Center Kansas City, KS United States; ^2^ Health Services and Outcomes Research Children's Mercy Kansas City Kansas City, MO United States; ^3^ Kenya Medical Research Institute Nairobi Kenya; ^4^ School of Medicine University of Missouri–Kansas City Kansas City, MO United States; ^5^ School of Pharmacy University of Missouri–Kansas City Kansas City, MO United States; ^6^ Global Health Innovations Dallas, TX United States

**Keywords:** early infant diagnosis (EID), HIV/AIDS, eHealth, mHealth, implementation science, Kenya

## Abstract

**Background:**

Literature suggests that electronic health (eHealth) interventions can improve the efficiency and accuracy of health service delivery and improve health outcomes and are generally well received by patients; however, there are limited data on provider experiences using eHealth interventions in resource-limited settings. The HIV Infant Tracking System (HITSystem) is an eHealth intervention designed to improve early infant diagnosis (EID) outcomes among HIV-exposed infants.

**Objective:**

We aimed to compare provider experiences with standard EID and HITSystem implementation at 6 Kenyan hospitals and 3 laboratories. The objective of this study was to better understand provider experiences implementing and using the HITSystem in order to assess facilitators and barriers that may impact adoption and sustainability of this eHealth intervention.

**Methods:**

As part of a randomized controlled trial to evaluate the HITSystem, we conducted semistructured interviews with 17 EID providers at participating intervention and control hospitals and laboratories.

**Results:**

Providers emphasized the perceived usefulness of the HITSystem, including improved efficiency in sample tracking and patient follow-up, strengthened communication networks among key stakeholders, and improved capacity to meet patient needs compared to standard EID. These advantages were realized from an intervention that providers saw as easy to use and largely compatible with workflow. However, supply stock outs and patient psychosocial factors (including fear of HIV status disclosure and poverty) provided ongoing challenges to EID service provision. Furthermore, slow or sporadic internet access and heavy workload prevented real-time HITSystem data entry for some clinicians.

**Conclusions:**

Provider experiences with the HITSystem indicate that the usefulness of the HITSystem, along with the ease with which it is able to be incorporated into hospital workflows, contributes to its sustained adoption and use in Kenyan hospitals. To maximize implementation success, care should be taken in intervention design and implementation to ensure that end users see clear advantages to using the technology and to account for variations in workflows, patient populations, and resource levels by allowing flexibility to suit user needs.

**Trial Registration:**

ClinicalTrials.gov NCT02072603; https://clinicaltrials.gov/ct2/show/NCT02072603 (Archived by WebCite at http://www.webcitation.org/71NgMCrAm)

## Introduction

In East Africa, electronic health (eHealth) interventions are increasingly being integrated into health care settings to support a range of HIV-related services and improve patient outcomes. In Kenya, where approximately 7000 children were infected with HIV in 2015 [1], we piloted an eHealth intervention called the HIV Infant Tracking System (HITSystem). The HITSystem aims to retain mother-infant pairs through the 18-month early infant diagnosis (EID) process or until an infant is identified as positive and treatment is initiated. The EID provider initiates enrollment in the HITSystem at the first EID visit, but laboratory technicians and mothers/caregivers are also active in the process. Algorithms driven by the infant’s date of birth and age-specific EID guidelines generate electronic alerts to inform clinical providers when a patient misses a key EID service and clinical and laboratory providers when polymerase chain reaction (PCR) sample processing is delayed. Additionally, the HITSystem sends short message service (SMS) texts to mothers alerting them when results are available at the hospital and serving as a reminder or cue to action for upcoming retesting services or when any service is missed. These processes are automated and may result in up to 14 provider alerts per infant enrolled (average of 3 to 5) and 3 or more SMS messages to mothers over the course of EID services, depending on infant test results and timeliness of seeking care.

Pilot data suggest that the HITSystem improves retention in EID services at 9 months, decreases turnaround times for PCR sample processing and mother notification of infant’s result, and increases the proportion of HIV-positive infants who achieve timely antiretroviral therapy (ART) initiation [2]. To date, over 10,000 mother-infant pairs have enrolled in the HITSystem at 30 hospitals in Kenya since its introduction in 2011. Hospitals using the HITSystem display moderate to high levels of ownership and adoption of the innovation [3]. A randomized controlled trial (RCT) is underway at 3 intervention and 3 control hospitals to rigorously evaluate the impact of the HITSystem on EID outcomes in Kenya [NCT02072603] [4].

While many eHealth interventions have reported on health outcomes [4-10] and patient acceptability or satisfaction [11-15] in Eastern Africa, few have reported extensively on the provider experience [16,17]. Health care providers are the key to the development and implementation of many eHealth interventions; thus, understanding their experience is critical in assessing factors that may facilitate or impede sustainable integration of new eHealth interventions into health care systems. This study describes the results from qualitative interviews conducted with hospital and laboratory providers involved in EID service provision as part of the ongoing RCT to evaluate the HITSystem [4]. The objective of this study was to better understand provider experiences implementing and using the HITSystem in order to assess facilitators and barriers that may impact the adoption and sustainability of this eHealth intervention. We drew upon constructs from implementation [18], technology acceptance [19,20], and diffusion models [21], which suggest that perceived usefulness and perceived ease of use play significant roles in a person’s intention to use a technology.

## Methods

### Study Setting

This process evaluation using qualitative methods was embedded within the RCT to evaluate the HITSystem [4]. The evaluation used a cluster-randomized design at 6 government hospitals (3 intervention, 3 control) and the designated central laboratories where study hospitals send their EID samples for processing. Hospitals were matched on hospital level (provincial, county, or district), geographic region, resource level, and patient volume. Intervention sites began using the HITSystem in February 2014. Clinicians at intervention hospitals and laboratory technicians at the central laboratories received a full day training on how to use the HITSystem, with periodic refresher training provided as needed by the site coordinator. Site coordinators were stationed at intervention hospitals to support HITSystem implementation. While providers from control hospitals and laboratories had no experience using the HITSystem, they were aware of the intervention and its functions. A total of 690 HIV-exposed infants were enrolled during the study period (392 at the intervention hospitals and 298 at the control hospitals).

### Study Participants and Procedures

Between February 2015 and January 2016, we conducted semistructured individual interviews with all key EID providers or HITSystem users at each of the study sites (total of 17 people). Control site clinicians and laboratory technicians were included to contextualize standard EID services during the time period of study implementation, allowing for better comparison to HITSystem-supported EID. The distribution of providers by site designation (intervention or control), facility type (hospital or laboratory), and participant role can be found in Table 1.

Interview guides were developed collaboratively by the Kenyan and US primary investigators, piloted among Kenyan-based site coordinators, and then refined based on feedback. Guides for control hospitals and laboratories assessed the perceived importance of EID, strengths and challenges associated with standard EID services, and hospital-laboratory communication under standard EID conditions. Guides for intervention hospitals and laboratories assessed differences in EID services since HITSystem introduction, challenges with HITSystem implementation, adequacy of HITSystem training, and mothers’ responses to the HITSystem.

**Table 1:** Description of participants.

Participant ID	Site designation and facility type	Facility ID	Infants enrolled (n)	Provider role
1	Control hospital	CH_A	115	Mentor mother^a^
2	Control hospital	CH_A	115	Mentor mother
3	Control hospital	CH_B	84	Nurse
4	Control hospital	CH_B	84	Mentor mother
5	Control hospital	CH_B	84	Nurse
6	Control hospital	CH_C	99	Mentor mother
7	Control hospital	CH_C	99	Mentor mother
8	Control laboratory	CL_D	—	EID^b^ data clerk^c^
9	Control laboratory	CL_D	—	EID data clerk
10	Control laboratory	CL_E	—	EID data clerk
11	Intervention hospital	IH_F	109	Mentor mother
12	Intervention hospital	IH_F	109	Nurse
13	Intervention hospital	IH_G	226	Nurse
14	Intervention hospital	IH_H	57	Mentor mother
15	Intervention laboratory	IL_I	—	Laboratory technician^c^
16	Intervention laboratory	IL_I	—	Laboratory technician
17	Intervention laboratory	IL_J	—	Laboratory technician

^a^Mentor mothers are HIV-positive mothers who have been through early infant diagnosis (EID) and provide a range of services including case finding and referral; defaulter tracing; case management; facilitation of support groups; health education; and support for enrollment, retention, and adherence.

^b^EID: early infant diagnosis.

^c^We interviewed laboratory personnel involved with EID-related documentation and hospital communication. At control laboratories, EID data clerks were responsible for manual EID documentation, and at intervention laboratories, laboratory technicians were responsible for HITSystem use (documentation and alert tracking).

All interviews were conducted by the study manager, who was trained in qualitative methods and human subject research. Written informed consent was obtained from participants prior to each interview. Interviews lasted approximately 45 minutes, were conducted in English, and occurred in a private setting. All study procedures were approved by the institutional review boards at the Kenya Medical Research Institute and University of Kansas Medical Center.

### Analysis Strategy

All interviews were audiorecorded, transcribed, and analyzed using Dedoose analysis software (SocioCultural Research Consultants). During the first round of coding, transcripts were coded independently by 3 study team members based on a priori and emergent themes [22]. These initial thematic codes were then combined into axial codes through group consensus [23,24]. In consultation with literature on facilitators and barriers related to provider adoption of mHeath interventions [18-21], we identified our axial codes closely related to the concepts of perceived usefulness and ease of use. To present the themes of our data, we discuss how the perceived usefulness and ease of use of the HITSystem serve as facilitators and barriers to HITSystem use.

## Results

### Facilitators to HIV Infant Tracking System Use

#### Perceived Usefulness

**Figure 1:**
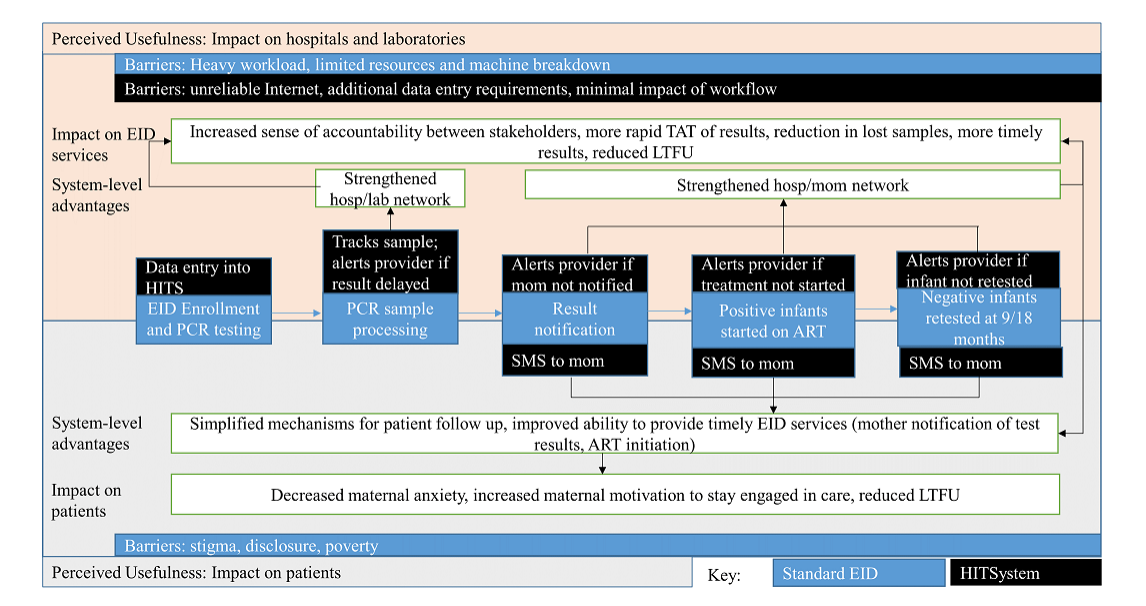
Perceived usefulness of the HIV Infant Tracking System (HITSystem). ART: antiretroviral therapy; EID: early infant diagnosis; LTFU: loss to follow up; PCR: polymerase chain reaction; SMS: short message service; TAT: turnaround time.

The perceived usefulness of the HITSystem was a strong theme among intervention respondents, who saw it as providing several benefits over standard EID care (Figure 1). Users described the primary uses of the HITSystem as (1) improving communication between mothers, clinicians, and laboratories and (2) simplifying mechanisms of patient follow-up. Together, these increased provider ability to deliver timely and efficient EID to their patients.

##### Improved Mother-Clinician and Clinician-Laboratory Communications

Clinicians emphasized the necessity of communication with mothers throughout the EID cascade: when laboratory results are ready to be picked up, follow-up services are required, or scheduled services are missed.

Clinicians found the HITSystem-driven automation of communication to mothers when results were ready useful for providing timely EID services to patients because:

...immediately when the results enter the HITSystem, the same system communicates to the mother. So the results can come in today, and the mother comes tomorrow. So there is a lot of improvement.

Participant 13

Clinicians noted the benefit to mothers, and as one remarked, “[mothers] are happy about being reminded” (Participant 13). While clinicians indicated that the HITSystem’s automated messages helped facilitate timely service provision, they still used existing methods of communication (phone calls or physical patient tracing) when patients did not return for services after receiving a text message, mothers did not have phones, or an incorrect phone number had been recorded.

Respondents also felt that the HITSystem was useful in facilitating communication between clinicians and laboratory technicians. In the absence of the HITSystem, provider-initiated phone calls were the most common method of communication between hospitals and laboratories, and clinicians expressed frustration with lack of clarity regarding lost or delayed sample processing in the laboratory. Control site providers expressed desires for improved systems of sample tracking in order “to assist us with follow-up and PCR results so we get them and the mothers can know the result” (Participant 6). Likewise, laboratory technicians from control sites described repeated communication regarding improper sample collection and shipping procedures which contributed to delayed turnaround time. One control site technician, for example, requested a Web-based system similar to the HITSystem to help address some of these challenges.

Introduce a system where the communication between us and them, it doesn’t need for them to pick up the phone call and start calling... If it was possible for them to actually have a Web-based system where they can get back to us, we can get back to them on and off, continuously because Web-based is real time...

Participant 9

Respondents also noted increased efficiency in hospital-laboratory communication with the HITSystem’s electronic messaging. The HITSystem’s sample tracking allowed clinicians to know when sample processing was delayed so that:

...we are not relying on them [laboratory personnel] much but on HITSystem. Unless there is a sample that was rejected, or was not sent, then we know faster through the HITSystem.

Participant 12

Providers also reported that the number of unreturned results had decreased because “even when the hard copy gets lost, the soft copy has been reported on” (Participant 14). The improved flow of information along with the HITSystem’s sample tracking led to an increased sense of accountability among laboratory technicians. This increased follow-up appeared to motivate increased prioritization and more efficient processing of EID samples.

But after the HITSystem, I personally realized there are some people who are concerned with this [turnaround time of EID results]. So it gave us a challenge to attend to this much faster. To me, this is like supervision, because things like seeing things are not going well, my colleague asks what is [taking] long. That is part of supervision. But previously you were just left to work, things go awry, and nobody asks.

Participant 16

With increased engagement facilitated by the HITSystem, providers described more options for hospital-laboratory communication and increased responsiveness to queries.

[Communication between hospital and laboratory] is better than before. My colleague just writes an email, it reaches the other colleague on the other side and we receive the results the same day. Before I would call with the only available contact, and find the staff is away on leave, or they request you call another day. So it was a problem.

Participant 13

These noted improvements contributed to a reduction in the frequency of phone calls required between the hospital and laboratory, for example, from “in a week, even more than 10 times” (Participant 12) to “maybe it’s one [call] a month, depending on what you’re following” (Participant 13).

##### Simplified Mechanisms for Patient Tracking

Clinicians from intervention sites discussed how the HITSystem simplified patient tracking. Clinicians from both intervention and control hospitals identified patient loss to follow-up as a barrier to providing comprehensive EID services, noting that in some instances, “you can meet with the mother, but she won’t bring the baby back to EID” (Participant 2). Clinicians from control sites described a manual tracking system that involved paper record review and required coordination between the maternal and child health departments (where mother-infant pairs present for care), internal hospital laboratories (where samples are drawn and shipped and results are received), and comprehensive care centers (where positive infants receive HIV care including ART initiation) for identifying and initiating follow-up with mothers whose infants had missed a critical service.

Should there be any defaulter, we have a diary ... and actually it is very, very full. She takes the contacts of all those babies who are exposed. She has the contact for the mothers so we are able to track until 18 months to 2 years ... and there is somewhere that she writes those who have defaulted and then been found.

Participant 4

Clinicians from intervention hospitals appreciated both the HITSystem-generated alerts for faster and easier identification of patients who missed services and the HITSystem’s facilitation of text messages for patient follow-up; clinicians attributed reduced rates of loss-to-follow-up to these advantages.

If we said today we stop this system, it would be bad, because we would see defaulters. When my colleague notices someone has missed their appointment, we find their contact and reach out to them to come for their appointment.

Participant 14

##### Improved Ability to Provide Timely and Efficient Early Infant Diagnosis Services

The benefits the HITSystem offered in sample tracking and result distribution allowed clinicians to provide more timely results for their patients. In the absence of the HITSystem, clinicians from control sites expressed frustration with long turnaround times for EID results and emphasized how these delays caused maternal anxiety and discouragement, impacted patient management and, for positive infants, delayed initiation of life-saving ART.

If they return and the results are not available, the mother is anxious to know the result which is not there. So convincing her, and telling her when the exact day the results will be available is a challenge, because she might lose hope.

Participant 2

You find that the result stays, and when the mother returns the next visit, results are not yet back, so the third time she returns then the results might be back. And sometimes the baby is weak, we suspect the baby is positive but you cannot start him on the medication until the [dried blood spot] is out.

Participant 5

The HITSystem was seen as an effective way of minimizing long turnaround time, reducing maternal anxiety, and increasing mothers’ motivation to stay engaged in EID:

...because once they get results early they are encouraged to come. When they come at 9 months, they complete 18 months without missing an appointment.

Participant 12

With more timely results, clinicians:

...noticed that [mothers] are happier that their baby’s sample is collected fast, results are back faster, and they know the results quickly.

Participant 11

Many clinicians also noted that HITSystem-driven improvements in turnaround time and return of results contributed to earlier initiation of ART among infants with a confirmed positive diagnosis.

Since HITSystem came, compared to the past, we get results faster, they are ready. When the mother [brings baby for testing], samples go, and she receives a message after 2 weeks or 3 weeks. Results are back and she gets results for the baby. Not like the past it would take 2 months, the mother will get discouraged and might be out there with a positive diagnosis and get lost. But HITSystem has helped a lot, even if the baby is positive it helps to initiate treatment early to prevent the baby from falling ill.

Participant 12

#### Ease of Use

Themes related to ease of use fell into 2 primary categories: complexity of the intervention and compatibility with existing workflow and patient needs.

##### Complexity

According to respondents, the HITSystem had a low level of complexity. Despite varied comfort levels with computers, most providers considered the HITSystem to be “straightforward, it is not something complicated” (Participant 16) and indicated that “if you’re trained, it [HITSystem] is easy” (Participant 12).

##### Compatibility

The HITSystem was largely perceived as compatible with current EID service delivery systems, with about half of intervention site clinicians stating that the HITSystem either improved or did not interfere with workflow, “because it is a touch of a button. I see that it will reduce our work load” (Participant 11). Likewise, laboratory technicians recognized that improved efficiencies offset any additional time expended on HITSystem-specific data entry, saying that:

...it takes time, but that is part of the work. And I know that when this comes, when all sites are there, it makes my work easier. So I do not take it as extra work.

Participant 16

Providers felt that the intervention was sensitive to and compatible with patients’ right to confidentiality throughout the EID process. They indicated that the automated HITSystem messages were informative enough to signal action without compromising the woman’s confidentiality or putting her at risk of unintentional disclosure.

Those messages are good; they don’t have any details about health. Just “bring your baby to the clinic.” Not about medication, so there is that confidentiality...Even the spouse is surprised, and she can tell him, “it’s immunization.” So he would not know anything, but if the message mentioned testing for HIV, it would arise suspicion.

Participant 14

### Barriers to HIV Infant Tracking System Use

#### Perceived Usefulness

While, overall, participants felt that the HITSystem was useful, many suggested adaptations to allow the HITSystem to better support their needs. These included suggestions to (1) “begin with the mother, since [antenatal care]...” (Participant 11) and support provision of prenatal care and HIV services throughout pregnancy, (2) increase the HITSystem’s capacity to “generate reports, depending on what the government wants” (Participant 17), and (3) integrate the HITSystem “with the [electronic medical record], with the [laboratory systems], so once the results have been posted, it will communicated with HITS so you don’t have to do double work” (Participant 17).

#### Ease of Use

HITSystem compatibility was strained by limited staffing and space for confidential enrollment.

For example, today, I had 2 clients, 1 old case, 1 new case. So you can’t take too much time, because you have 1 room, and sister needs to use it. So it becomes a challenge. Even one of them ran away because [there is no privacy].

Participant 12

Half of the clinicians indicated that entering data into the HITSystem during the patient encounter was:

...slightly difficult because you are entering this and that. There’s also another one waiting for counseling, another one wants something about adherence, so you find it taking a bit longer.

Participant 14

Therefore, some clinicians stated that on busy days:

...sometimes you find yourself pushing the HITSystem to another day, depending on the workload...maybe I will do it another day when I don’t have a heavy workload.

Participant 13

HITSystem use was also dependent on reliable internet connectivity and adequate internet and phone credit, which both clinicians and laboratory technicians identified as barriers to consistent use:

We are not connected to the network. We use the modems. Yes? Well if we run out of credit that is the end of the story.

Participant 15

While providers from intervention hospitals and laboratories appreciated the continuous on-the-job training they received from study staff, most felt that additional, off-site training would be beneficial to minimize distractions, enhance skills, and ensure continued implementation, especially as updates were made to the HITSystem.

It’s good also to have a repeat of the same [training] because...as time goes by, I think you people are improving on the system and maybe some new ideas have also been included up to now because people keep on growing. And I believe it’s growing.

Participant 15

Furthermore, there were challenges impacting standard HIV services that continued to hinder EID service provision that the HITSystem was unable to address. Lack of consistent resources was cited as a key challenge to providing timely and efficient EID services at both intervention and control sites. Laboratory technicians’ ability to achieve quick turnaround time, even with HITSystem tracking and prompts, was dependent on working machines and availability of laboratory supplies. Laboratory technicians recognized that:

...once in a while we have delays and mostly it is because of lack of reagents or breakdown of equipment.

Participant 17

Likewise, for hospitals, stock outs for sample collection materials were a challenge:

...because you find the mother will come and be asked to return next week or the following day because the kits are finished.

Participant 2

Clinicians from intervention and control hospitals also discussed nondisclosure of HIV status and poverty as barriers to providing EID services. Providers described how adhering to medication and appointment schedules, breastfeeding, and family planning recommendations may be difficult for undisclosed mothers. They indicated that hospital transport expenses were challenging for women and that women may arouse suspicion if they ask partners or family members for transport money. Respondents indicated that, in some cases, women may be forced to choose between using money for transport to the hospital or other essentials. One intervention clinician indicated that mothers were sometimes concerned about receiving a message asking them to return to the hospital when they didn’t have money for transport. In these situations, clinicians would:

...assure them that the SMS is just to prepare you, and once you get fare you can come to the clinic expecting the results to be ready.

Participant 13

## Discussion

### Principal Findings

Previous research on implementation of new technological interventions in health care has identified perceived usefulness and ease of use as 2 of the most important factors impacting provider adoption [25]. Our findings suggest that providers see both of these factors as prominent features of the HITSystem, suggesting perceived usefulness and ease of use may have facilitated the high adoption at the intervention sites. Strengthened networks between key stakeholders through improved mechanisms for patient tracing, sample tracking, and result distribution and automated communication between stakeholders allowed providers to provide more efficient EID services. The intervention’s compatibility with existing workflows further facilitated HITSystem adoption by providers. Providers indicated that these advantages contributed to noticed improvements in EID services including shortened turnaround time of HIV DNA PCR sample processing, reductions in lost samples, reduced maternal anxiety, lower rates of loss to follow-up, more rapid initiation of ART among infants identified as positive, and improved communication between stakeholders.

Barriers to intervention implementation included shortages of HITSystem-specific resources including reliable internet and phone credit and the time associated with additional data entry requirements in light of heavy workloads. Providers described how they were able to overcome these barriers and integrate the HITSystem with minimal disruptions to their daily working routines by delaying data entry until their workload was lighter or until the internet was more reliable. Overall, interviews indicated that intervention providers felt that HITSystem-driven improvements in EID service provision outweighed the challenges associated with HITSystem use. Even after HITSystem implementation, however, barriers at the system level (supply stock outs and machine breakdowns) and patient level (maternal poverty and fear of disclosure) remained beyond the scope of the HITSystem intervention and continued to hinder timely and complete EID services.

Responsiveness to feedback generated during these interviews will improve the utility of the HITSystem within implementing hospitals and laboratories and can generate additional provider buy-in, facilitate greater HITSystem implementation, and support sustainability. After providers’ suggestions, a new module of the HITSystem that tracks women throughout their pregnancy and then automatically links the infant to the EID was developed and rolled out to all facilities implementing the HITSystem [26]. Linkage of the HITSystem with other national systems, including the Laboratory Information Management System, and development of enhanced reporting features to generate customized data reports are actively underway. These linkages are expected to decrease data entry requirements for clinicians and laboratory technicians, further enhancing compatibility of the HITSystem. Additional training will be provided to clinical and laboratory staff using the HITSystem to ensure they are familiar with and comfortable using these additional features.

### Limitations

Our study had several limitations that should be noted. First, while we interviewed the primary EID providers and HITSystem users at control and intervention hospitals, our sample of only a few facilities is relatively small and limited our ability to achieve saturation on all emergent themes of interest. Second, as part of the study, each intervention site had a designated study coordinator to support HITSystem implementation. Thus, these data may not reflect the range of provider experiences in programmatic settings. Third, interviews were conducted around the time study participants were returning for their 9-month services. Since the intervention lasts through completion of EID at approximately 18 months, these data do not capture the full cycle of implementation through the EID cascade. Lastly, other factors outside of the scope of providers likely impact eHealth adoption. For example, other studies have shown that cost to the institution or other stakeholders [25,27], stakeholder collaboration, and government involvement can also impact eHealth adoption [25,28]. Our study was limited in that we interviewed only providers and thus were unable to assess factors at different levels that may facilitate or impede adoption, sustainability, and scalability.

### Conclusions

Despite these limitations, this study provides a comprehensive qualitative evaluation of provider experiences using an eHealth intervention in the context of an RCT, illustrating how implementation research can complement RCTs. The noted advantages that the HITSystem offered over standard EID services, allowing providers to better meet patient needs, were a strong theme across site and provider type and served as a facilitator to HITSystem uptake. Workload and resource constraints were barriers to use. Provider experiences with the HITSystem indicate that the usefulness of the HITSystem, along with the ease with which it can be incorporated into hospital workflows, contribute to its sustained adoption and use in Kenyan hospitals. Their perspective suggests that other eHealth interventions in similar settings may maximize the potential for implementation success by assuring end users see clear advantages of the new technology and can easily integrate it into their workflow. Care should be taken in intervention design to account for variations in workflows, patient populations, and resource levels by allowing for flexibility in implementation to suit varying workflow and user needs. However, a larger sample of stakeholders (patients, hospital administrators, policy makers, government representatives, etc) is needed to more comprehensively describe the multilevel factors that may influence adoption and sustainability.

## Abbreviations

ART: antiretroviral therapy
EID: early infant diagnosis
HITSystem: HIV Infant Tracking System
PCR: polymerase chain reaction
RCT: randomized controlled trial
SMS: short message service
